# Editorial: Epigenetics in Heart Failure Developing: The Orchestra of Etiology and Comorbidities

**DOI:** 10.3389/fcvm.2022.869613

**Published:** 2022-03-18

**Authors:** Alexander E. Berezin, Ioana Mozos, Daniel Petrovič

**Affiliations:** ^1^Internal Medicine Department, Zaporozhye State Medical University, Zaporozhye, Ukraine; ^2^Department of Functional Science, Victor Babes University of Medicine and Pharmacy, Timisoara, Romania; ^3^Faculty of Medicine, Institute of Histology and Embryology, University of Ljubljana, Ljubljana, Slovenia

**Keywords:** heart failure, epigenetic mechanisms, co-morbidities, biomarkers, management, diagnosis, prognosis

This editorial summarizes the contributions to the Frontiers Research topic “*Epigenetics in Heart Failure Developing: The Orchestra of Etiology and Comorbidities*.”

Heart failure (HF) is a multimodal condition associated with a wide range of comorbidities and increased mortality, and remains a major clinical, public health, and economic problem despite significant efforts to improve management of the disease (1). There is resoundingly clear scientific proof of the fact that a number of new cases of HF can be explained by cumulative effects of coexisting diseases involving overlapping genetic and epigenetic mechanisms (2). Current understanding of the basic principles of the molecular machinery underlying occurrence of HF plays a pivotal role in precise care of HF and management of regenerative and translational therapies (3). Indeed, epigenetic influences, being crucial post-transcriptional triggers, are considered to be powerful regulators of biomechanical and oxidative stresses, systemic and microvascular inflammation, myocardial fibrosis, and endothelial dysfunction (4). Moreover, key epigenetic mechanisms are regarded as promising molecular targets for new therapeutic interventions in HF including cardiac reprogramming, inducible progenitor cell transplantation, stimulation of resident cardiomyocytes differentiation and proliferation, and inhibition of cardiomyocyte apoptosis (5). These approaches are expected to be effective tools to manage HF at the different stages of its natural evolution.

This Research Topic aimed to explore epigenetics and genetic regulation in the development of heart failure along with an elucidation of the role of epigenetic biomarkers and new epigenetic biomarker-based models that could be applied for the prediction of HF and point-of-care management of the disease.

The narrative review by Berezin and Berezin depicted a pivotal role of circulating extracellular vesicles (EVs) in pathogenesis of different phenotypes of HF. Being a cargo for a broad spectrum of regulatory peptides, factors of coagulation, lipids, proteins, growth factors, active molecules, hormones, chromatin, and micro-RNAs (mi-Rs), EVs that originated from several parental cells (cardiac myocytes, mature and progenitor endothelial cells, mononuclear cells, platelets, and skeletal muscle cells) are embedded onto tissue reparation, cell proliferation and differentiation, angiogenesis, and neovascularization, whereas depending on apoptotic transformation of the parental cells, proteomics/lipidomics, and also a signature of mi-Rs of EVs these particles were found to be inductors of both direct and indirect tissue damage. Authors evaluated resoundingly clear scientific proof that affected a link between metabolic comorbidities, altered cardiac and vascular reparation, a risk of HF manifestation, and progression in connection with a profile of circulating EVs as a biomarker of certain epigenetic response. Moreover, the signature of EVs in peripheral blood is considered to be not only powerful diagnostic and prognostic biomarkers of HF, but also a promising target for translational therapy in the future.

Another study by Peterlin et al. presents the results of a systematic review in which authors evaluated miRs profiles in HF patients and control individuals that were responsible for several signaling pathways, such as interleukin-2 signaling and p53 activity regulation mechanisms, the MAP kinase/PI3 kinase-Akt signaling, apoptosis and angiogenesis pathways, including transforming growth factor-beta-related mechanism. The study was based on searching for articles in the PubMed database. Authors found that from 72 previously selected potential molecular candidates only five miRs, namely miR-1228, miR-122, miR-423-5p, miR-142-3p, and exosomal miR-92b-5p, were differentially expressed in HF patients and that this signature of miRs seems to be a prognostic biomarker in the clinical setting.

Chen et al. focused on the impact of estimated glomerular filtration rate (eGFR) on the amount of adverse cardiovascular (CV) outcomes in patients with heart failure with preserved ejection fraction (HFpEF). They used a database of 3,392 subjects who were enrolled in the TOPCAT (Treatment of Preserved Cardiac Function Heart Failure with an Aldosterone Antagonist) trial and examined clinical outcomes (all-cause death, CV death, and HF hospitalization) by multivariable Cox regression models. Authors showed that eGFR 30-59 ml/min/1.73 m^2^ was strongly associated with an increased risk of all-cause death (adjusted hazard ratio [HR] = 1.47; 95% confidence interval [CI] = 1.24–1.76; *P* < 0.001), CV death (adjusted HR= 1.53; 95% CI = 1.23–1.91; *p* < 0.001), and HF hospitalization (adjusted HR = 1.21; 95% CI = 1.00–1.47; *p* = 0.049) in HFpEF patients.

The study by Chuda et al. was performed with the aim of elucidating the relationship of dehydration, body mass index (BMI), hemodynamics, cardiopulmonary exercise test (CPET) parameters, 6 min walk distance (6MWD), and Kansas City Cardiomyopathy Questionnaire (KCCQ) score with the occurrence of atrial fibrillation (AF) in patients hospitalized due to advanced HF. Authors found that AF patients were more dehydrated, had significantly lower exercise capacity, lower KCCQ overall summary score, and higher New York Heart Association class than those with sinus rhythm. Higher BMI, higher left atrial volume index, lower tricuspid annular plane systolic excursion, and lower percentage of total body water were considered to have been independently related to AF in patients with HF.

The narrative review by Leczycki et al. related to potential prognostic factors that may predict clinical outcomes in adults with congenital heart diseases. They evaluated numerous potential biomarkers reflecting biomechanical stress obtained by echocardiography and magnetic resonance imaging, cardiopulmonary exercise testing, and circulating biomarkers including natriuretic peptides, growth-differentiation factor 15, high-sensitivity troponin T, galectin-3, angiopoietin-2, asymmetrical dimethylarginine, and high-sensitivity C-reactive protein. Authors compared several biomarkers' models and found them quite promising, although a multiple biomarker panel seems to be much more effective.

In conclusion, epigenetic regulation of the HF development was found to be crucially important in understanding new tools for diagnosis and medical care of the disease. Although these interventions seem to be quite futuristic, they continue to attract the attention of numerous researchers due to recent discoveries in the subtle molecular mechanisms of regulation of the phenotypic response of myocardium. Large clinical trials are needed to accelerate the implementation of these innovative approaches that are considered to be much more than promising in the context of developing concise diagnostic tools, restoring adverse cardiac remodeling, and reversing HF.

## Author Contributions

AB wrote the article text and evaluated benefits of the articles of the research issue. IM and DP checked the proof and provided appropriate corrections. All authors were guest associate editors of the Research Topic and edited the text.

## Conflict of Interest

The authors declare that the research was conducted in the absence of any commercial or financial relationships that could be construed as a potential conflict of interest.

## Publisher's Note

All claims expressed in this article are solely those of the authors and do not necessarily represent those of their affiliated organizations, or those of the publisher, the editors and the reviewers. Any product that may be evaluated in this article, or claim that may be made by its manufacturer, is not guaranteed or endorsed by the publisher.
